# Polysulfone Membranes: Here, There and Everywhere

**DOI:** 10.3390/membranes16010035

**Published:** 2026-01-05

**Authors:** Pere Verdugo, Iwona Gulaczyk, Magdalena Olkiewicz, Josep M. Montornes, Marta Woźniak-Budych, Filip F. Pniewski, Iga Hołyńska-Iwan, Bartosz Tylkowski

**Affiliations:** 1 Eurecat, Centre Tecnològic de Catalunya, Chemical Technologies Unit, Marcel·lí Domingo 2, 43007 Tarragona, Spain; magda.olkiewicz@eurecat.org (M.O.);; 2 Faculty of Chemistry, Adam Mickiewicz University in Poznań, ul. Uniwersytetu Poznańskiego 8, 61-614 Poznań, Poland; iwona.gulaczyk@amu.edu.pl; 3 NanoBioMedical Centre, Adam Mickiewicz University in Poznan, Wszechnicy Piastowskiej 3, 61-614 Poznań, Poland; marta.budych@amu.edu.pl; 4 Faculty of Oceanography and Geography, University of Gdańsk, Al. Piłsudskiego 46, 81-378 Gdynia, Poland; filip.pniewski@ug.edu.pl; 5 Department of Pathobiochemistry and Clinical Chemistry, Faculty of Pharmacy, Ludwik Rydygier Collegium Medicum in Bydgoszcz, Nicolaus Copernicus University in Toruń, 85-094 Bydgoszcz, Poland; igaholynska@cm.umk.pl; 6 Department of Clinical Neuropsychology, Faculty of Health Science, Ludwik Rydygier Collegium Medicum in Bydgoszcz, Nicolaus Copernicus University in Toruń, ul. Sklodowskiej Curie 9, 85-094 Bydgoszcz, Poland

**Keywords:** polysulfone, modified polysulfone, gas separation, water purification

## Abstract

Polysulfone (PSU) membranes are widely recognized for their thermal stability, mechanical strength, and chemical resistance, making them suitable for diverse separation applications. This review highlights recent advances in PSU membrane development, focusing on fabrication techniques, structural modifications, and emerging applications. Phase inversion remains the predominant method for membrane synthesis, allowing precise control over morphology and performance. Functional enhancements through blending, chemical grafting, and incorporation of nanomaterials—such as metal–organic frameworks (MOFs), carbon nanotubes, and zwitterionic polymers—have significantly improved gas separation, and water purification., In gas separation, PSU-based mixed matrix membranes demonstrate enhanced CO_2_/CH_4_ selectivity, particularly when integrated with MOFs like ZIF-7 and ZIF-8. In water treatment, PSU membranes effectively remove algal toxins and heavy metals, with surface modifications improving hydrophilicity and antifouling properties. Despite these advancements, challenges remain in optimizing cross-linking strategies and understanding structure–property relationships. This review provides a comprehensive overview of PSU membrane technologies and outlines future directions for their development in sustainable and high-performance separation systems.

## 1. Introduction

Polysulfones are polymers composed of aryl and sulfonyl groups. They are typically synthesized via the polycondensation of bisphenol A and dichlorodiphenyl sulfone. The diphenyl sulfonyl moiety contributes high bond dissociation energy, resulting in excellent tensile strength and resistance to oxidizing agents. These properties can be further enhanced by reinforcing the polymer with glass or carbon fibers. Polysulfones were first synthesized in 1959, presented during a conference in 1965, and their commercialization under the trade name Udel started in 1966 by Union Carbide. They were the first thermoplastics specifically engineered for prolonged use at temperatures exceeding 150 °C [1]. Polysulfones exhibit relatively high glass transition temperatures (Tg), typically ranging from 190 °C to 240 °C, ensuring structural integrity from −100 °C to 150 °C. These polymers are amorphous, semi-transparent, and possess low creep, excellent electrical insulating properties, and self-extinguishing behavior. Their high chemical resistance is largely attributed to the diphenyl sulfonyl moiety, which enhances oxidative stability through electron delocalization via resonance [2]. Mechanically, polysulfones exhibit high tensile strength, 70–75 MPa; Young’s modulus, 2.5–2.6 GPa, and excellent impact resistance [3]. However, prolonged outdoor exposure makes polysulfones susceptible to chemical degradation due to their aromatic ether backbone. This vulnerability can be mitigated by incorporating carbon fillers, applying surface coatings, or metalizing the material. Despite these measures, polysulfones remain generally unsuitable for long-term outdoor use. Ultraviolet (UV) radiation and moisture can lead to the breakdown of polymer chains, causing irreversible deterioration of the material’s structural and functional properties over time. Additionally, polysulfones are not resistant to low-polarity organic solvents such as ketones, chlorinated hydrocarbons, and aromatic hydrocarbons.

**Figure 1:**
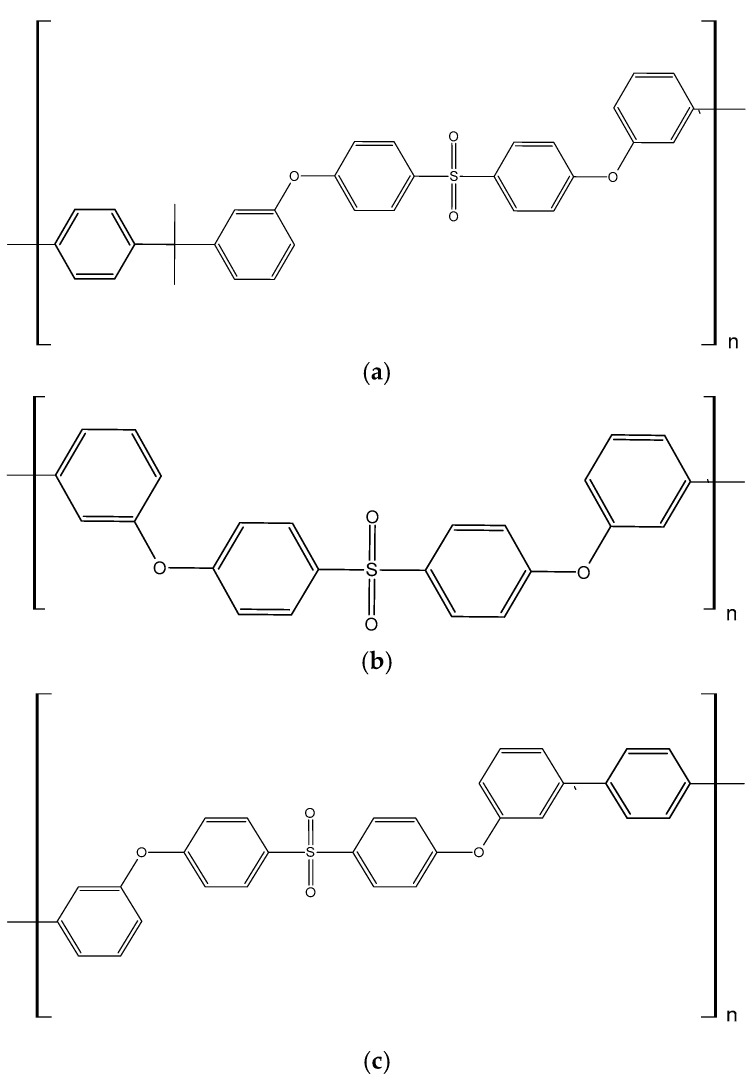
Chemical structures of (**a**) polisulfone (PSU), (**b**) poliethersulfone (PES) and (**c**) polyphenylene sulfone (PPSU).

Currently, three primary variants of polysulfones are in use: polysulfone (PSU), polyethersulfone (PES), and polyphenylene sulfone (PPSU). These variants differ in their chemical structures, particularly in the functional groups located between adjacent benzene rings (see Figure 1).

While polysulfone-based thermoplastics exhibit broadly similar performance characteristics, their structural variations confer distinct properties that render each variant more suitable for specific applications. Among these, polysulfone (PSU) is recognized as the most economically viable, which has led to its prominence in both research and industrial modification efforts. In 2024, the global market valuation for PSU was approximately USD 1.55 billion. Forecast models project continued growth, with the market expected to expand from USD 1.62 billion in 2025 to USD 2.36 billion by 2034, corresponding to a compound annual growth rate (CAGR) of approximately 4.30% over the forecast period (2025–2034) [4].

## 2. Preparation and Modification of Polysulfone Membranes

Polysulfone (PSU), polyethersulfone (PES), and polyvinylidene fluoride (PVDF) are among the most widely utilized conventional polymeric materials for the fabrication of membranes and membrane elements [5]. The morphology of polymeric membranes, defined by the structure and spatial arrangement of pores as well as other microstructural features, is a critical determinant of their performance across various applications.

The phase inversion precipitation, also referred to as non-solvent induced phase separation (NIPS), is among the most commonly used techniques for the fabrication of polysulfone membranes at both laboratory and industrial scales. In this process, a polymer solution undergoes liquid–liquid or liquid–gas demixing, resulting in the formation of a solid membrane. This method was pioneered in the late 1950s by Sidney Loeb (University of California) and Srinivasa Sourirajan (University of Florida) for the development of reverse osmosis membranes [6,7]. Since its development, phase inversion has been regarded as one of the most versatile and straightforward techniques for the fabrication of polymeric membranes [8,9].

**Figure 2:**
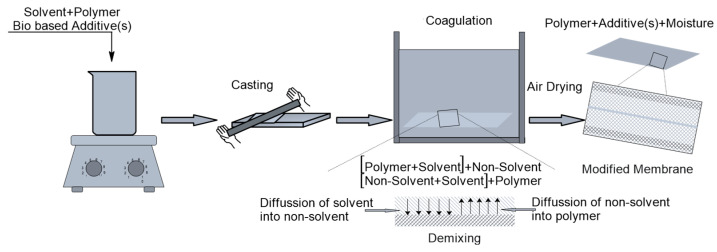
Membrane preparation by the phase inversion precipitation method.

As illustrated in Figure 2, the process begins with the dissolution of a polymer in an appropriate solvent to form a homogeneous dope solution. This solution is subsequently cast and immersed in a non-solvent bath, initiating a diffusion-driven exchange: the non-solvent permeates into the polymer film while the solvent diffuses outward. This exchange induces phase separation, resulting in the formation of a porous membrane structure. The polymer-rich phase solidifies to form the continuous matrix of the membrane, whereas the non-solvent-rich phase gives rise to the pore network. Following phase separation, the membrane is typically subjected to washing and air-drying to remove residual solvent and to stabilize the final structure [10].

**Figure 3:**
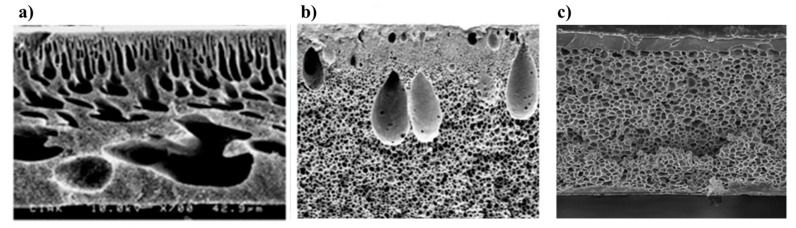
Morphologies of PSU membrane with: (**a**) finger-like structure [18], (**b**) sponge-like [19] and (**c**) sponge-like with macrovoids [20] structures.

A critical factor in the development and application of PSU membranes is the precise control of their polymeric morphology, which plays a pivotal role in determining membrane performance. Morphological characteristics can vary significantly depending on parameters such as polymer concentration, the choice of solvent/non-solvent system, and processing conditions. Key structural features—including pore size, surface porosity, and the presence of macrovoids—govern membrane morphology and directly influence performance metrics such as permeability and fouling resistance. Enhancements in these features typically lead to improved membrane efficiency and reduced fouling propensity. Numerous techniques and strategies for tailoring the morphology of PSU membranes have been extensively studied, with the effects of morphological modifications on mechanical integrity, chemical stability, operational performance, and fouling behavior thoroughly investigated. These findings are detailed in the following articles [11,12,13,14,15,16,17]. As a matter of example, Figure 3 provides SEM micrographs of polysulfone membranes with different morphologies. Figure 3a demonstrates PSU membrane with a finger-like structure [18], while Figure 3b,c show PSU membranes with sponge-like [19] and sponge-like with macrovoids [20] structures, respectively.

In addition to structural optimization, PSU membranes can be functionally enhanced through blending or chemical modification. Blending involves the incorporation of other polymers or inorganic materials into the PSU matrix to tailor its physicochemical properties and improve performance in specific applications such as water purification, gas separation, and food processing. Chemical functionalization, on the other hand, facilitates the introduction of reactive groups onto the polymer backbone, thereby enhancing membrane selectivity, hydrophilicity, antifouling characteristics, and thus expanding the potential range of membrane applications [21].

**Figure 4:**
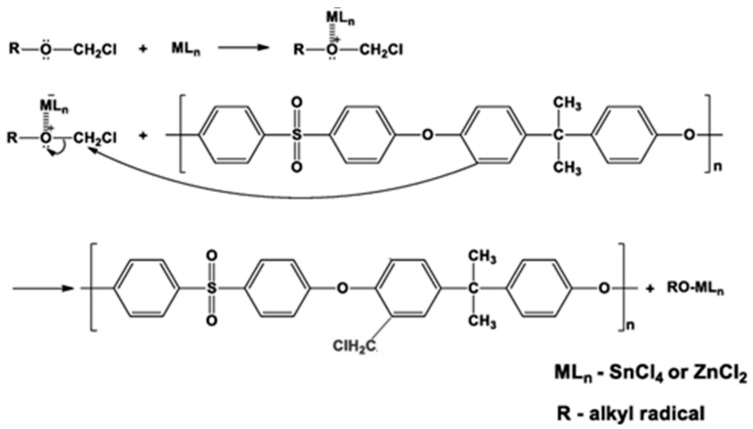
Chloromethylation mechanism of polysulfone with chloromethyl alkyl ethers.

In the case of polysulfone, functionalization can be achieved via two primary approaches: copolymerization with functionalized comonomers or post-polymerization modification. The copolymerization route often results in low-molecular-weight polymers due to the reduced reactivity of certain comonomers during condensation polymerization [22]. Moreover, some functional groups may be incompatible with the harsh polymerization conditions typically required for nucleophilic aromatic substitution, which generally involves elevated temperatures (>150 °C) and prolonged reaction times (>12 h). Alternatively, post-functionalization (a strategy broadly used for PSU) allows for the introduction of various functional groups after polymer synthesis. Common post-functionalization methods include sulfonation, bromination, amidoalkylation, lithiation, and chloromethylation, each offering distinct advantages in tuning the polymer’s chemical, thermal, and separation properties. It is important to highlight that chloromethylation reactions have been extensively applied as effective strategies for generating reactive intermediates, which can be further functionalized through a variety of synthetic pathways tailored to specific applications. This utility stems from the high reactivity and versatility of the chloromethyl group (─CH_2_Cl), which readily undergoes nucleophilic substitution with diverse functional groups. The reaction proceeds via an electrophilic aromatic substitution mechanism and typically involves chlorinated hydrocarbons (e.g., chloroform, dichloroethane, trichloroethane) as solvents, along with chloromethyl alkyl ethers such as monochloromethyl methyl ether, bis(chloromethyl) methyl ether, 1,4-bis(chloromethoxy)butane, and 1-chloromethoxy-4-chlorobutane as chloromethylating agents. A halogen-containing Lewis acid catalyst, such as SnCl_4_ or ZnCl_2_, is required to facilitate the reaction (Figure 4) [23,24,25]. The effectiveness of a series of Lewis-type catalysts on the substitution degree has been studied in literature, and reported [12] that it decreases in the order: SnCl_4_ > ZnCl_2_ > AlCl_3_ > TiCl_4_ > SnCl_2_ > FeCl_3_.

**Figure 5:**
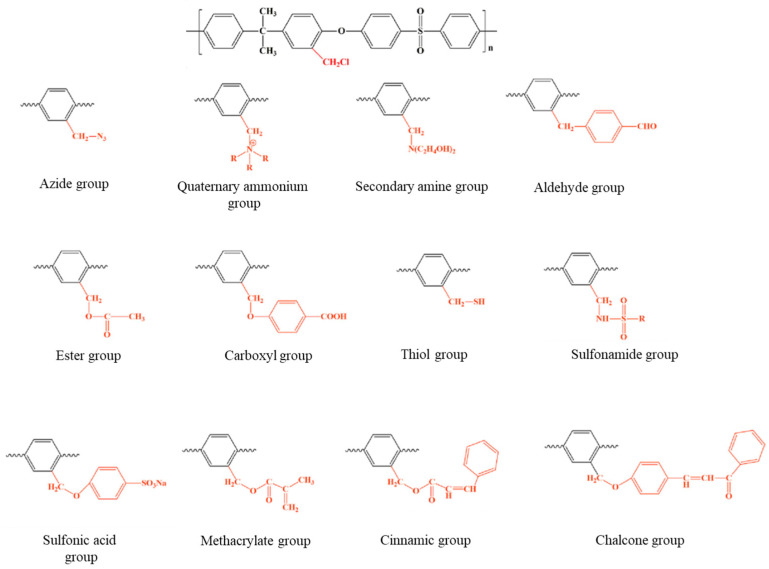
Polysulfones functionalization using chloromethylated precursors.

In the case of PSU, the chloromethyl cation preferentially reacts with the electron-rich aromatic rings of the bisphenol A unit, resulting in the grafting of chloromethyl groups. The reaction exhibits high regioselectivity due to the electron-donating effects of the oxygen atoms, which activate specific positions on the aromatic rings. In contrast, the aromatic rings within the arylsulfone unit are deactivated by the strongly electron-withdrawing sulfone group. Consequently, the chloromethylation of polysulfone yields a derivative containing a maximum of two chloromethyl groups per repeating unit, reflecting the influence of the sulfone group’s deactivating effect. To minimize undesirable side reactions and obtain soluble chloromethylated polysulfones (CMPSU) with a high degree of substitution, investigators have emphasized the importance of maintaining specific reaction conditions. These include: (I) a low concentration of polysulfone in solution (typically 2% *w*/*v*); (II) extended reaction times ranging from 28 to 140 h; (III) a high molar ratio between the polysulfone repeating unit and the chloromethylating agent (commonly 1:10:10); and (IV) a limited amount of catalyst, typically between 0.10 and 0.18 mmol of SnCl_4_ [12,26]. The primary advantage of polysulfone functionalization using chloromethylated precursors lies in the facile substitution of chloromethyl groups with a wide range of nucleophilic agents, as illustrated in Figure 5. Consequently, polysulfones incorporating chloromethyl functionalities within their backbone play a pivotal role in broadening their application spectrum by enabling structural diversification and the development of novel properties.

## 3. Application of Polysulfone and Polysulfone Modified Membranes

### 3.1. CO_2_ Capture and Gas Separation

Addressing global warming driven by anthropogenic CO_2_ emissions remains a critical challenge in contemporary environmental research. Conventional liquid solvent-based absorbents for CO_2_ capture, while effective, are hindered by high operational costs and complex handling requirements, limiting their scalability for industrial applications. In contrast, solid sorbents derived from earth-abundant materials present a promising alternative, offering reduced energy demands for CO_2_ desorption and improved process efficiency.

Nogalska et al., inspired by nature, designed and developed artificial stomata for carbon dioxide absorption based on a polysulfone membrane contactor [27]. By using the phase inversion precipitation method and varying membrane preparation parameters (like different solvents, coagulation bath composition, and casting knife thickness), the authors successfully fabricated membrane contactors with thicknesses ranging from 76 to 176 µm and different morphologies, from spongy-like to open microvoid structures. The authors reported that the highest CO_2_ absorption flux (67.5 mmol/m^2^·s) was achieved using a fringelike macrovoid membrane, which could absorb more CO_2_ than natural stomata (40 µmol/m^2^·s). The aforementioned results presented the first membrane contactor established for ambient CO_2_ capture, and indicated that CO_2_ flux increased with the absorbent flow rate over the range of liquid velocities investigated. The flux grew with the increase in membrane macrovoid size, due to the decrease in membrane mass transfer resistance.

As reported by Chukov et al. [3] and Nisar et al. [28], the mechanism of CO_2_ capture in PSU-based membranes was primarily governed by their distinctive structural characteristics—most notably, the presence of polar sulfone (SO_2_) groups and a rigid aromatic polymer backbone. These features facilitated selective dipole–quadrupole interactions with CO_2_ molecules, while also imparting long-term thermal and mechanical stability to the membrane. CO_2_ absorption into the membrane material followed Henry’s law, with solubility generally increasing under higher pressures and lower temperatures. Once absorbed, CO_2_ diffuses through the membrane as a result of concentration gradients. The polymer’s free volume—defined as the intermolecular space between polymer chains—played a critical role in gas diffusion, with larger free volumes enabling more rapid transport. Membrane thickness also influenced diffusion rates; thinner membranes can enhance permeance [29].

Recently, Abdulabbas and co-workers [30] addressed a research gap by analyzing the influence of various PSU membrane designs and operational parameters on gas separation performance. Utilizing computational fluid dynamics (CFD), the authors demonstrated several key findings: (i) increasing the gas flow rate from 20 to 75 mL/min resulted in a 27% enhancement in the mole fraction of CO_2_ in the permeate stream; (ii) elevating the operating temperature from 313 K to 393 K led to an 8% reduction in CO_2_ mole fraction; and (iii) increasing the feed pressure from 3 to 9 bar yielded a 12.5% increase in CO_2_ mole fraction. These results underscore the importance of optimizing both membrane configuration and operating conditions to enhance the separation efficiency of CO_2_/CH_4_ mixtures. Table 1 provides experimental data on permeability and selectivity values for CO_2_/CH_4_ separation using PSU and PSU composite membranes reported in the literature.

Almuhtaseb et al. [36] systematically investigated the suitability of two solvents: chloroform (CF) and tetrahydrofuran (THF) for casting PSU membranes intended for the separation of individual components from a binary CO_2_/CH_4_ gas mixture. The influence of feed gas pressure on membrane performance was examined across a range of 1–10 bar, with particular emphasis on permeability and selectivity. The authors reported that the maximum CO_2_ and CH_4_ permeabilities for membranes fabricated using THF were 62.32 and 2.06 Barrer at 1 and 2 bar, respectively. In comparison, membranes cast with CF exhibited maximum permeabilities of 57.59 and 2.12 Barrer at 1 and 2 bar, respectively. Selectivity values at 1 bar were 48 for THF and 36 for CF. Moreover, the results demonstrated a consistent decline in both permeability and selectivity with increasing pressure for membranes prepared with either solvent. Notably, PSU membranes synthesized using THF outperformed those fabricated with CF, particularly when benchmarked against the Robeson upper bound. These findings underscore the critical role of solvent selection during membrane fabrication for gas separation.

To further enhance CO_2_ separation can be modulated by incorporation of different fillers/additives, such as ionic liquids, zeolites, metal nanoparticles, silica-based materials, carbon molecular sieves, and metal–organic frameworks (MOFs).

The 2025 Nobel Prize in Chemistry was awarded to Susumu Kitagawa, Richard Robson, and Omar M. Yaghi for their work on developing metal–organic frameworks. MOFs are crystalline porous materials composed of metal ions or clusters coordinated to organic linkers, forming highly tunable structures with large surface areas and high adsorption capacities [37], making them useful for applications like gas storage, carbon capture, and catalysis. The organic linkers within MOFs promote favorable interactions between the polymer matrix and the filler, improving compatibility and dispersion [35]. The high permeability and selectivity observed in MOF-containing PSU membranes primarily stem from the significantly greater adsorption affinity of MOFs for CO_2_ compared to CH_4_. In certain MOFs, molecular sieving also contributes to enhanced selectivity, as their pore sizes can be precisely tailored to fall between the kinetic diameters of the target gas molecules. This enables preferential diffusion of smaller CO_2_ molecules while restricting the passage of larger CH_4_ molecules, thereby improving separation performance [38]. Among MOFs, Zeolitic Imidazolate Frameworks (ZIFs)—including ZIF-7, ZIF-8, ZIF-11, ZIF-90, ZIF-71, ZIF-108, and ZIF-3025—have demonstrated promising performance in CO_2_/CH_4_ separation due to their tailored pore structures and chemical stability. Zeolitic Imidazolate Framework-7 (ZIF-7) is a porous crystalline material composed of zinc ions coordinated with benzimidazole ligands, forming a sodalite (SOD) topology. Its intrinsic pore size is approximately 3.0 Å. The benzimidazole ligand exhibits a strong affinity for CO_2_, which induces a gate-opening phenomenon: the angle between the vertical plane separating the interior of the ZIF-7 structure and the ligand increases from the conventional 48° to approximately 61–62°, thereby expanding the pore size up to 5.2 Å [39]. Cacho-Bailo et al. [40] prepared a PSU hollow-fiber membrane with ZIF-7, and they reported that CO_2_/CH_4_ gas mixture separation should be carried out at 4 bar and at lower temperatures. By performing high-temperature tests of H_2_/CO_2_ separation using ZIF-7 membranes, the authors observed that CO_2_ is preferentially adsorbed within the porous structure of ZIF-7 at lower temperatures. As temperature increases, adsorption effects diminish, and diffusion becomes the dominant transport mechanism. Under these conditions, the H_2_/CO_2_ separation factor increased to 5.7 at 150 °C, with H_2_ permeance reaching a value seven times higher than that observed at 35 °C. According to the authors, this separation factor, which slightly exceeds the theoretical Knudsen selectivity, indicates that CO_2_ transport through the ZIF-7 structure is not significantly restricted under elevated temperature and pressure conditions.

Negi and Suresh [35] reported that ZIF-8 exhibits excellent permeability and selectivity for CO_2_/CH_4_ separation. The enhanced CO_2_ permeability in ZIF-8 is attributed to its higher adsorption affinity for CO_2_ compared to CH_4_. Additionally, its pore size of approximately 3.4 Å lies between the kinetic diameters of CO_2_ (3.3 Å) and CH_4_ (3.8 Å), enabling effective molecular sieving and selective transport of CO_2_. The authors investigated dense polysulfone membranes with ZIF-8 loadings ranging from 0.5 to 5 wt % for the separation of CO_2_ from both synthetic CO_2_/CH_4_ mixtures. They reported that the membrane containing 1 wt % ZIF-8 exhibited the greatest improvement, with CO_2_ permeability and CO_2_/CH_4_ selectivity increasing approximately 58% and 41%, respectively, compared to the pristine PSU membrane, as it is shown in Table 1. Furthermore, the authors demonstrated that at higher filler loadings, ZIF-8 particles tended to agglomerate, which may reduce their effectiveness and hinder membrane performance. In tests with mixed gases, CO_2_ permeability increased by approximately 8% to 34%, depending on the gas composition. Notably, the enhancement was more pronounced in mixtures with lower CO_2_ concentrations. Nordin et al. [41] reported that for asymmetric PSU membranes, an optimal ZIF-8 loading is 0.5 wt %. Their results demonstrated that at this loading, CO_2_ permeance increased by 37% (from 21.27 GPU to 28.22 GPU), and CO_2_/CH_4_ selectivity improved from 19.43 to 23.19 compared to the neat PSU membrane. These findings highlight the effectiveness of low ZIF-8 concentrations in enhancing membrane performance without inducing particle agglomeration or compromising structural integrity. Sorribas et al. [42] incorporated silica–ZIF-8 composite spheres into a polysulfone matrix and observed a 300% increase in CO_2_ permeability compared to pristine PSU membranes, highlighting the potential of hybrid membrane systems for enhanced gas separation. Similarly, Sasikumar et al. [43] incorporated amine-functionalized ZIF-8 into PSU hollow-fiber membranes, achieving a remarkable 61.46% increase (from 13.78 to 22.25) in CO_2_/CH_4_ selectivity compared to pristine PSU membranes.

Jonnalagedda and Kuncharam investigated the influence of indium-based 2D and 3D MOFs—MIL-68(In)–NH_2_ and In(aip)_2_—embedded within PSU matrices for gas separation applications [44]. Their findings revealed maximum CO_2_/CH_4_ selectivities of 19.8 and 24.4 for mixed matrix membranes containing 15 wt % MIL-68(In)–NH_2_ and 10 wt % In(aip)_2_, respectively. Additionally, Khan et al. synthesized and incorporated polyethyleneimine (PEI)-functionalized bimetallic MOFs, specifically PEI@HKUST-1(Cu, Mg), into PSU membranes. Their results indicated that a 5 wt % loading of this MOF filler significantly improved CO_2_/CH_4_ selectivity [45].

Ionic liquids (ILs) are organic molten salts with melting points below 100 °C. Room-temperature ionic liquids (RTILs) are a subset of ILs that remain liquid at temperatures below ambient conditions (T_m_elting < 373 K). These compounds exhibit a range of remarkable properties that set them apart from conventional liquids, including negligible vapor pressure, high thermal stability, non-flammability, a liquid range extending up to at least 300 °C, and excellent solubility for a wide variety of inorganic and organic compounds. Furthermore, their physicochemical properties can be precisely tuned for specific chemical applications through the appropriate selection of the anion, cation, and substituents on the cationic component [46]. Alkhouzaam et al. [47] investigated CO_2_/CH_4_ separation using dense polysulfone-supported ionic liquid membranes. The authors blended four ionic liquids (ILs)—1-alkyl-3-methylimidazolium bistriflamide ([C_4_mim][NTf_2_]), diisopropyl 1-alkyl-3-methylimidazolium bistriflamide ([DIP-C_4_mim][NTf_2_]), tributylmethylphosphonium formate ([P_4441_][formate]), and tributylmethylammonium formate ([N_4441_][formate])—with polysulfone to produce functional dense polymer-supported IL membranes (DPSILMs). According to the reported results, the DPSILMs exhibited clear chemical and physical changes in the PSU structure and demonstrated good IL distribution within the polymer matrix. The highest CO_2_/CH_4_ selectivities reported were 70, 63, 47, and 32 for PSU-2.5 wt% [C_4_mim][NTf_2_], PSU-2.5 wt% [DIP-C_4_mim][NTf_2_], PSU-0.5 wt% [N_4441_][formate], and PSU-5 wt% [P_4441_][formate], respectively. Moreover, the authors reported, that all ILs improved CO_2_/CH_4_ selectivity compared to pure PSU, as it is showed in Table 1. The effect of IL concentration on separation was also examined: selectivity was inversely proportional to IL concentration, with the highest values observed at lower IL loadings. Stability tests showed minimal loss of [N_4441_][formate] and [P_4441_][formate] compared to literature reports, while no loss of [C_4_mim][NTf_2_] and [DIP-C_4_mim][NTf_2_] was detected. Thus, the synthesized DPSILMs are stable under high pressure for extended periods.

Nisar et al. [48] developed and evaluated nanoarchitectured composites based on PSU and carbon-based fillers with magnetically responsive properties for efficient CO_2_ capture. The fillers—carbon nanotubes (CNTs) and activated carbon (AC) embedded with iron nanoparticles—were incorporated into the PSU matrix at concentrations ranging from 5 to 20 wt %. Transmission electron microscopy (TEM) confirmed a uniform dispersion of the fillers within the polymer matrix, with particle sizes between 47 and 54 nm. Thermal analysis revealed an increase of approximately 4 °C in both the onset (T_onset_) and maximum (T_max_) degradation temperatures upon the addition of 5 wt % nanoparticles, compared to the pristine PSU. Differential scanning calorimetry (DSC) showed that the glass transition temperature (T_g_) remained unchanged across all formulations. Increasing the filler content led to a gradual reduction in water contact angle values, indicating enhanced hydrophilicity of the nanocomposites. The CO_2_ capture capacity of the PSU-based nanocomposites ranged from 40 to 61 mg CO_2_/g at 45 °C, significantly surpassing that of the unmodified PSU. This performance represents a notable advancement over previously reported systems, establishing a new benchmark for polymer-based CO_2_ sorbents.

Ahn et al. [49] developed a polysulfone-based mixed-matrix membrane containing dispersed nonporous fumed silica nanoparticles. During these studies permeabilities of H_2_, He, O_2_, N_2_, CH_4_, and CO_2_ were measured as a function of silica volume fraction, and diffusion and solubility coefficients were determined via the time-lag method. Experimental results revealed that the influence of silica on gas permeability deviates from predictions based on the Maxwell model: O_2_ permeability increased approximately fourfold, and CH_4_ permeability more than fivefold compared to pristine PSU. However, the permeability–selectivity trade-off for CO_2_/CH_4_ remained aligned with the upper-bound trend as silica content increased, giving the following selectivity values 6.5, 6.7, 7.0, 5.7 and 6.0 for membranes containing 0 wt% Si, 0.05 wt% Si, 0.10 wt% Si, 0.15 wt% Si and 0.25 wt% Si, respectively, at 4.4 atm and 35 °C. The observed enhancement in gas permeability is attributed to increased free volume arising from inefficient polymer chain packing and additional voids at polymer–silica interfaces. Larger gas molecules exhibited greater permeability enhancement, primarily due to increased diffusion coefficients, which concurrently reduced pure-gas selectivity. Consequently, incorporation of fumed silica did not surpass the upper-bound performance limit, yet significantly disrupted polymer packing, generating free volume in low-free-volume glassy polymers to a greater extent than in high-free-volume analogues.

Chemical modification of the polymer backbone or side chains of PSU, particularly through halogenation and fluorination, has also been shown to significantly enhance gas separation performance. These modifications improve key membrane properties such as gas permeability, selectivity, and resistance to plasticization. Halogenation involves the introduction of halogen atoms (e.g., fluorine or chlorine) into the PSU backbone or pendant groups, thereby altering the membrane’s physicochemical interactions with specific gas molecules and improving separation selectivity. As an example, Chinese patent CN107376603B claims application of PSU membranes or chloromethylated polysulfone membranes for CO removal in hydrogen production tail gas.

Although the membrane-based gas separation industry has been established for nearly 45 years, there remains a strong impetus to develop advanced polymer membranes with enhanced resistance to plasticization and physical aging. Cross-linked polymer structures have emerged as a promising strategy to address these challenges, offering improved mechanical and chemical stability. However, current cross-linking approaches face several unresolved limitations, including substantial reductions in gas permeability due to densification of the membrane matrix, restricted tunability of the cross-linked architecture, and an incomplete understanding of structure–property relationships, which hinders predictive design of high-performance membranes [50]. In the case of cross-linked PSU membrane development for gas separation, chloromethylated polysulfone could be used as a starting polymer.

### 3.2. Water Purification

Water pollution has emerged as a critical global issue, driven not only by the worldwide expansion of industrial activity but also by the increasing demand for clean and safe drinking water. The presence of hazardous contaminants, ranging from heavy metals and organic pollutants to biologically derived compounds and emerging contaminants, further exacerbates this challenge. The scarcity of potable water amplifies the impact of contamination, making water pollution one of the most pressing environmental and public health concerns.

In response, researchers are actively pursuing innovative and sustainable solutions for effective water treatment. PSU membranes have been widely employed in water remediation and wastewater treatment due to their favorable properties, including excellent chemical and thermal stability, high mechanical strength, and ease of structural modification [51]. Recently, in Asia, PSU membranes have been applied to face a challenge related to the mitigation of algal toxin release from algae-contaminated water sources, which poses a serious threat to drinking water safety and public health. Although algal cells and algal toxins differ significantly in size and solubility, they often coexist in micro-polluted water bodies, complicating conventional treatment approaches. To address this issue, Zhang et al. [52] investigated the use of PSU membranes for the simultaneous removal of algae and algal toxins from drinking water sources. Their study demonstrated that under an operating pressure of 0.05–0.08 MPa, PSU hollow-fiber membranes with a molecular weight cut-off (MWCO) of 200 kDa effectively removed algal cells, even at influent concentrations ranging from 1 to 30 cells/mL. Despite the widespread use of PSU in water treatment, its intrinsic hydrophobicity can negatively impact membrane performance, particularly in terms of fouling and permeability. Surface modification techniques allow for the tuning of physicochemical properties such as hydrophilicity and porosity, thereby enhancing membrane permeability and antifouling efficiency [53].

PSU and PSU-modified membranes have also been applied in membrane distillation (MD), which is a thermally driven separation process that utilizes differences in vapor pressure across a hydrophobic porous membrane to selectively transport water vapor, while effectively retaining non-volatile solutes [54]. MD has gained prominence in water desalination applications due to several key advantages: (a) lower operating temperatures compared to conventional thermal distillation; (b) reduced hydrostatic pressure requirements; (c) high solute rejection rates, theoretically up to 100% for non-volatile compounds; (d) less stringent mechanical demands on membrane materials; and (e) lower susceptibility to membrane fouling relative to pressure-driven processes such as reverse osmosis [55,56]. To date, a wide range of modifiers has been employed to enhance the performance of PSU membranes. These include inorganic nanomaterials such as halloysite nanotubes, titanium dioxide, alumina, silicon dioxide, manganese oxide, zirconia, carbon nanotubes, graphene oxide, zinc oxide, and MOFs. In addition to these cellulose nanocrystals or macromolecular modifiers—such as dendrimers, quaternary ammonium compounds, polyzwitterions, and chitosan—have emerged as promising candidates for membrane surface functionalization in water treatment applications. These materials have been the subject of numerous scientific and review articles due to their potential to improve membrane hydrophilicity, antifouling properties, and overall separation performance [51,57,58,59,60,61]. As an example, Zodrow et al. [62] incorporated nanosilver particles into the PSU membrane matrix to enhance hydrophilicity and mitigate biofouling and viral penetration. Using the phase inversion technique, Kang et al. [63] fabricated PSU membranes blended with sulfonated graphene oxide (SGO), achieving significantly improved hydrophilicity. Their findings revealed that the incorporation of 1.5 wt% SGO increased water flux by more than 125% compared to pristine PSU membranes. Similarly, Balakashrisna Prabhu et al. [64] employed a wet coagulation method to introduce a chitosan derivative, 1H-pyrazole-4-carbaldehyde, into the PSU matrix. The presence of hydrophilic functional groups (e.g., hydroxyl, amine, imine) markedly enhanced membrane wettability, reducing the contact angle from 70 ± 1° for unmodified PSU to 62 ± 1° for blended membranes, and increasing water flux from 24 L·m^−2^·h^−1^ to 351 L·m^−2^·h^−1^ at 0.8 MPa. Ravishankar et al. [21] incorporated graphene oxide (GO) nanoparticles into PSU to produce PSf/GO membranes, which exhibited superior hydrophilicity compared to neat PSU, with a water contact angle of 34.2° and permeability of 52.1 L·m^−2^·h^−1^·bar^−1^.

Chemical grafting using chloromethylated polysulfone as a precursor enables surface modification of PSU membranes through the attachment of grafting agents bearing functional end groups and long compatibilizing tails. Dong et al. [65] grafted poly[poly(ethylene glycol) methyl ether methacrylate], PPEGMA, and poly(glycidyl methacrylate), PGMA, onto chloromethylated polysulfone to enhance its antifouling properties. Literature also reports that chloromethylated polysulfone modified with grafted zwitterionic polymers exhibits superior antifouling performance compared to unmodified PSU [66].

In particular, Maggay et al. [67] demonstrated that biofouling of PSU membranes modified with a zwitterionic copolymer—comprising styrene and 4-vinylpyridine units via a dual-bath grafting procedure—was reduced by 87% relative to pristine PSU membranes. This significant reduction in biofouling highlights the potential of zwitterionic-modified PSU membranes not only for water purification applications but also for use in biomedical devices such as hemodialysis membranes.

## 4. Conclusions

Polysulfone (PSU) membranes remain integral to advanced separation technologies due to their outstanding thermal stability, mechanical robustness, and chemical resistance. Progress in fabrication methods, particularly phase inversion, has enabled precise control over membrane morphology, while chemical functionalization and nanocomposite strategies have substantially broadened their performance envelope. The incorporation of inorganic fillers, metal–organic frameworks (MOFs), and zwitterionic polymers has driven notable enhancements in gas separation and water purification.

PSU-based mixed-matrix membranes demonstrate superior selectivity and permeability for CO_2_/CH_4_ separations, whereas surface-modified variants exhibit improved antifouling characteristics and hydrophilicity in aqueous environments. 

Despite these advances, critical challenges persist, including plasticization resistance, physical aging, and limited tunability of cross-linked architectures. To address these issues, future research should focus on:

- Developing scalable and spatially controlled cross-linking strategies to mitigate physical aging without compromising permeability.
- Employing multi-scale modeling and molecular simulations to elucidate structure–property relationships and predict long-term performance.
- Designing hybrid membranes with hierarchical architectures to enhance selectivity and mechanical integrity under operational stresses.
- Exploring novel application domains such as selective ion separation, bio-hybrid systems, and membranes for energy storage and conversion.
- Advancing green and solvent-free fabrication techniques to improve sustainability and reduce environmental impact.

Such targeted efforts will accelerate the transition of PSU membranes toward next-generation, high-performance, and environmentally responsible separation systems.

## Figures and Tables

**Table 1:** Permeability and selectivity values for CO_2_/CH_4_ separation using PSU and PSU modified membranes reported in the literature.

Membrane	Mw (Da)	Solvent	Additive	CO_2_/CH_4_ Selectivity	CO_2_ Permeability (Barrer)	CH_4_ Permeability (Barrer)	∆P (bar)	T (°C)	Ref.
PSU	~22,000	tetrahydrofuran	-	50	30.0	0.65	1	20	[29]
PSU	~22,000	chloroform	-	35	24.8	0.72	1	20	[29]
PSU	~22,000	chloroform	-	25	6.9	0.28	10	22	[31]
PSU-0.5 wt% [C_4_mim][NTf_2_]	~22,000	chloroform	ionic liquid 1-alkyl-3-methylimidazolium bistriflamide ([C_4_mim][NTf_2_])	57	10.9	0.19	10	22	[32]
PSU-2.5 wt% [C_4_mim][NTf_2_]	~22,000	chloroform	ionic liquid 1-alkyl-3-methylimidazolium bistriflamide ([C_4_mim][NTf_2_])	70	11.5	0.16	10	22	[31]
PSU-0.5 wt% [DIP-C_4_mim][NTf_2_]	~22,000	chloroform	diisopropyl 1-alkyl-3-methylimidazolium bistriflamide ([DIP-C_4_mim][NTf_2_])	61	12.2	0.19	10	22	[31]
PSU-2.5 wt% [DIP-C_4_mim][NTf_2_]	~22,000	chloroform	diisopropyl 1-alkyl-3-methylimidazolium bistriflamide ([DIP-C_4_mim][NTf_2_])	63	13.8	0.22	10	22	[31]
PSU-5 wt% [P_4441_][formate]	~22,000	chloroform	tributylmethylphosphonium formate ([P_4441_][formate])	32	11.5	0.40	10	22	[31]
PSU-12.5 wt% [P_4441_][formate]	~22,000	chloroform	tributylmethylphosphonium formate ([P_4441_][formate])	31	17.3	0.48	10	22	[31]
PSU-0.5 wt% [N_4441_][formate]	~22,000	chloroform	tributylmethylammonium formate ([N_4441_][formate])	47	12.5	0.26	10	22	[31]
PSU-2.5 wt% [N_4441_][formate]	~22,000	chloroform	tributylmethylammonium formate ([N_4441_][formate])	46	10.2	0.22	10	22	[31]
PSU- wt32% ZIF-8	35,000	chloroform	32 wt% of MSS-Z8 synthesied by authors	32	24.4	-	1	35	[33]
PSU	35,000	chloroform	-	~31	6.1		1	35	[34]
PSU	77,000–83,000	chloroform	-	~22	-	-	-	-	[35]
PSU	77,000–83,000	chloroform	1 wt% of synthesied ZIF-8	~31	-	-	-	-	[35]

## Data Availability

No new data were created or analyzed in this study.
